# THE IMPACT OF COVID-19 ON SERVICE USAGE IN A RURAL “VILLAGE”

**DOI:** 10.1093/geroni/igac059.1779

**Published:** 2022-12-20

**Authors:** Joonyoung Cho, Ruth Dunkle, Julie Tarr

**Affiliations:** University of Michigan-Ann Arbor, Ann Arbor, Michigan, United States; University of Michigan, Ann Arbor, Michigan, United States; ShareCare of Leelanau, Leland, Michigan, United States

## Abstract

The Covid-19 pandemic disrupted service access and use for many older adults aging in place. This study focuses on understanding how a rural “Village”, where services are primarily provided by volunteers to older adults, adjusted to Covid-19. The sample is drawn from service users of at least one or more services between January and October 2020 (N=233). Survey and qualitative data were gathered via telephone-interview (N=80). This study examined (1) the impact of Covid-19 on service use and ability to stay at home, 2) services offered, and 3) changes to day to day life. In the sample, 53.75% is 80+ in age, 70% female, and 43.75% lived alone. 15% of respondents reported reduction in service used, 10% an increase, 65% no change, and 6.25% used no services. Some services such as rides to medical care, and grocery delivery increased, other services (e.g., caregiving) were reduced. 26.24% respondents believed that the organization’s services helped them stay at home during Covid-19. Other services were developed to better serve older adults such as phone reassurance and expansion of delivery of medication and food. Open ended responses identified how Covid-19 impacted day to day life. While many reported negative ways such as changing mental health perspectives and limiting medical care, others report life being more peaceful and providing time to enjoy nature. Findings provide an understanding that services while disrupted continued for one volunteer organization with some service users showing resilience in their day to day lives.

